# Lipemic Aqueous Humor in Hyperlipidemia and Diabetes Mellitus: A Case Report

**DOI:** 10.1155/crop/9931034

**Published:** 2026-04-23

**Authors:** Jing Wu, Jia Ying, Yu Zhu, Jun Li

**Affiliations:** ^1^ Department of Ophthalmology, Lishui Municipal Central Hospital, The Fifth Affiliated Hospital of Wenzhou Medical University, Lishui, China, wmu.edu.cn

**Keywords:** diabetes mellitus, hyperlipidemia, lipemic aqueous humor

## Abstract

**Introduction/Background:**

Lipemic aqueous humor is a rare ocular condition characterized by a milky appearance in the anterior chamber, primarily associated with hyperlipidemia and poorly controlled diabetes. This case highlights the diagnostic challenges and management of lipemic aqueous humor, emphasizing the need for differentiation from other etiologies such as infectious endophthalmitis.

**Case Presentation:**

A 26‐year‐old woman with type 1 diabetes and hyperlipidemia presented with unilateral vision loss and ocular redness. Examination revealed visual acuity of 20/400, conjunctival congestion, corneal clouding, and milky anterior chamber exudate. Laboratory tests showed severe hyperglycemia (fasting glucose 33.79 mmol/L) and hypertriglyceridemia (23.71 mmol/L). Initial management included anterior chamber irrigation and intravitreal antibiotics due to suspected infection. Treatment was augmented with corticosteroids, mydriatics, and systemic control of glucose and lipids. Visual acuity improved to 20/70 by Day 3 and recovered to 20/20 by Day 6, with stability at 1‐month follow‐up.

**Discussion/Conclusion:**

This case underscores that lipemic aqueous humor, though rare, should be considered in the differential diagnosis of anterior chamber opacities in patients with metabolic disorders. The disruption of the blood–aqueous barrier, potentially exacerbated by underlying inflammation in the context of hyperlipidemia and diabetes, is key to its pathogenesis. Prompt diagnosis, metabolic control, and targeted anti‐inflammatory therapy can lead to excellent visual outcomes, potentially avoiding unnecessary invasive interventions.

## 1. Introduction

Lipemic aqueous humor is an uncommon ocular manifestation characterized by the extravasation of lipoproteins into the anterior chamber, resulting in a milky‐white appearance. It is frequently linked to systemic conditions such as hyperlipidemia and poorly controlled diabetes mellitus. This case report describes a 26‐year‐old woman with type 1 diabetes and hyperlipidemia who presented with sudden vision loss due to lipemic aqueous humor. We aim to illustrate the clinical presentation, diagnostic challenges, and management approaches, highlighting the importance of differentiating this condition from other causes of anterior chamber opacities, including infectious endophthalmitis and inflammatory uveitis.

## 2. Case Report

**Figure 1:**
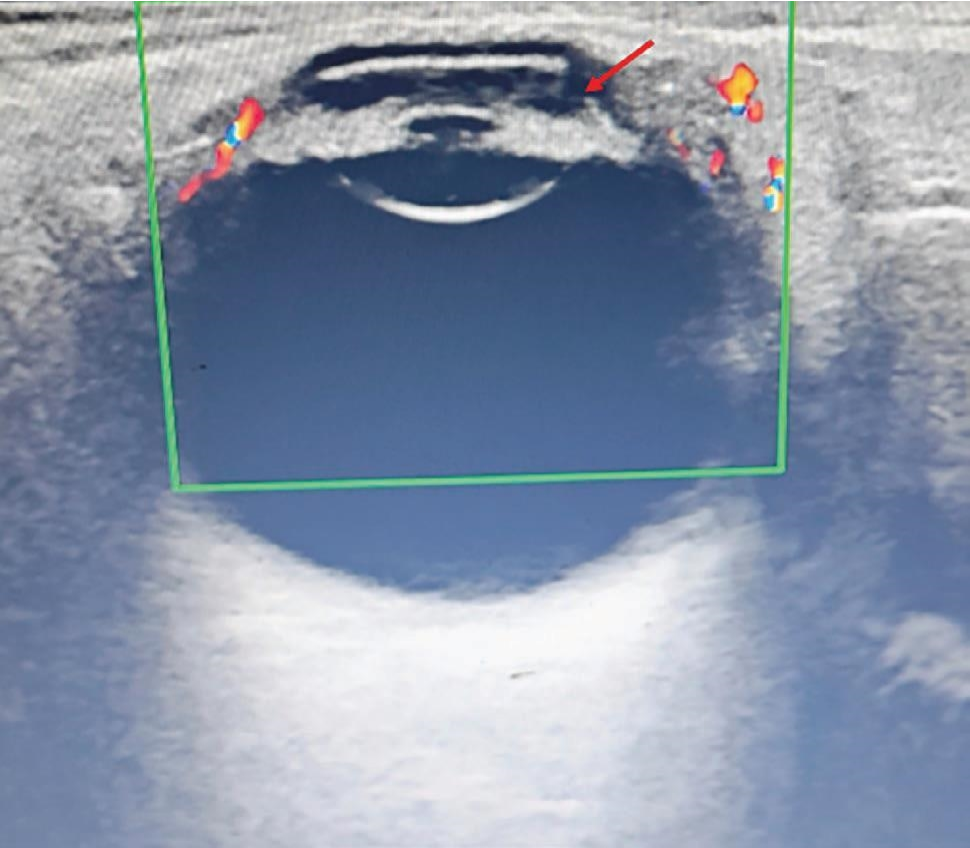
Ultrasound imaging of the left eye prior to emergency intervention. Note the presence of severe anterior chamber clouding (arrow in red) and the absence of significant vitreous abnormalities.

This case report has been prepared in accordance with the CARE guidelines. A 26‐year‐old woman presented to our ophthalmology clinic with a 3‐day history of redness and vision loss in her left eye. She had a 5‐year history of type 1 diabetes mellitus (T1DM) with suboptimal glycemic control. There was no history of ocular trauma or hereditary eye disease. Ophthalmologic examination revealed a best‐corrected visual acuity (BCVA) of 20/400 in the left eye and an intraocular pressure (IOP) of 12 mmHg. Slit‐lamp examination demonstrated mixed conjunctival congestion, corneal clouding, a milky‐white exudate in the anterior chamber, and iris adhesions. The posterior segment was not visible due to anterior opacities. Ocular ultrasound confirmed severe anterior chamber clouding without vitreous abnormalities (Figure 1). Laboratory investigations indicated fasting glucose (33.79 mmol/L), glycated hemoglobin (18.5%), total cholesterol (15.72 mmol/L), and triglyceride (23.71 mmol/L). White blood cell count, C‐reactive protein, and serum amylase were within normal limits. Abdominal ultrasound and a routine chest scan showed no obvious abnormalities. A blood smear revealed the presence of gram‐positive cocci. Given the presence of gram‐positive cocci on blood smear and the clinical picture, a preliminary diagnosis of acute infectious endophthalmitis was made.

**Figure 2:**
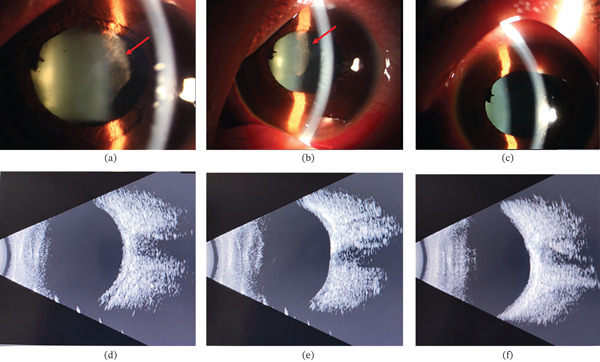
Sequential anterior segment photographs (top row) and corresponding ocular ultrasound images (bottom row) of the left eye during hospitalization. (a, d) Day 2: Partial clearance of anterior chamber opacities is observed (red arrow), with residual echoes visible on ultrasound. (b, e) Day 3: Marked resolution of anterior chamber clouding with further normalization of ultrasound findings. (c, f) Day 6: The anterior chamber appears clear, and ultrasound confirms the absence of significant pathological changes.

**Figure 3:**
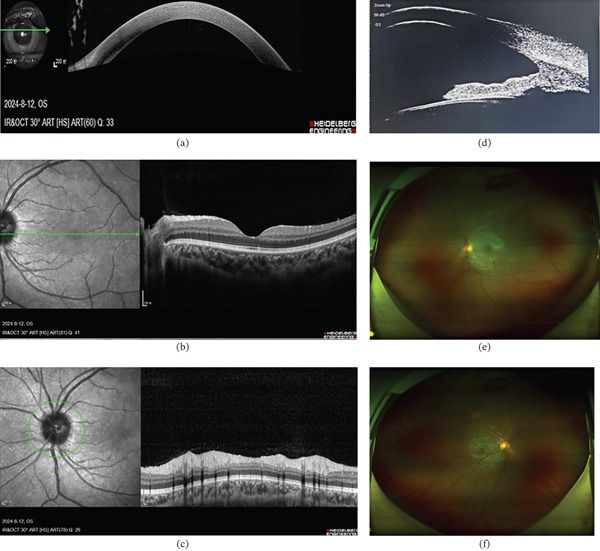
Multimodal imaging of the left eye at discharge (Day 6). (a) Anterior segment OCT and (d) UBM demonstrate persistent aqueous flare in the anterior chamber. (b) Macular OCT reveals intact retinal architecture without edema or structural disruption. (c) Optic nerve head OCT shows mild disc hyperemia. (e, f) Wide‐angle fundus photography confirms an unremarkable posterior pole and peripheral retina in both eyes.

Immediate anterior chamber irrigation and intravitreal injection of vancomycin hydrochloride (1 mg/0.1 mL) and ceftazidime were performed. Additional treatments included periocular methylprednisolone sodium succinate (40 mg), topical tobramycin–dexamethasone drops (0.1% four times daily), and atropine drops (1% three times daily), alongside systemic hypoglycemic and hypolipidemic medications. On Day 2, BCVA remained 20/400, but corneal transparency was improved, and anterior opalescent clouding markedly reduced, with minimal exudate adhered to the iris. The pupil was pharmacologically dilated, and the lens exhibited slight anterior capsule pigmentation. Fundus examination became possible, and ultrasound showed vitreous abnormalities (Figure 2). By Day 3, BCVA improved to 20/70, with clear cornea, aqueous flare (+), dilated pupil, clear lens, and mildly hyperemic optic disc (Figure 2). Aqueous humor cultures were negative, and subsequent blood cultures identified human *Staphylococcus*, which was considered a contaminant, systemic antibiotics were therefore discontinued. Upon discharge on Day 6, BCVA reached 20/20 in both eyes, and ocular conditions remained stable at 1‐month follow‐up (Figure 3). The final diagnoses were lipemic aqueous humor, T1DM, and hyperlipidemia.

## 3. Discussion

Hyperlipidemia contributes to various systemic and ocular complications. Chylous serum appears when plasma triglyceride concentrations exceed 1000 mg/dL [1], whereas lipemia retinalis (LR) typically manifests at levels above 2500 mg/dL [2, 3]. Notably, not all cases of severe hypertriglyceridemia present with LR, as its development is influenced by additional factors such as genetic predispositions, disorders, hematocrit levels, and vessel translucency [4, 5]. In our case, the patient′s triglyceride level was 23.71 mmol/L (2098.23 mg/dL), which was not associated with LR.

Lipemic aqueous humor is an exceedingly rare condition characterized by the presence of lipoproteins within the anterior chamber, resulting in a distinct milky‐white appearance [5]. Under physiological conditions, the aqueous humor is essentially devoid of lipoproteins. Chylomicrons, the largest lipoprotein particles (750–1000 nm), are notably smaller than blood cells and most plasma proteins. Their density closely approximates that of aqueous humor, allowing them to remain suspended following extravasation through a compromised blood–aqueous barrier (BAB). This barrier disruption, central to the process, may not solely result from the direct physicochemical effects of hyperlipidemia. An associated low‐grade inflammatory state, such as iridocyclitis, which can be triggered or exacerbated by metabolic dysregulation, may synergistically contribute to increased vascular permeability and BAB breakdown.

A key diagnostic challenge is to distinguish lipemic aqueous humor from other causes of anterior chamber turbidity. The Tyndall effect, caused by light scattering by elevated protein levels, characterizes intraocular inflammation and is clinically recognized as aqueous flare. This phenomenon typically indicates an inflammatory breakdown of the BAB, which is often accompanied by inflammatory cells. Similarly, hypopyon, which generally settles inferiorly at the 6 o′clock position in infectious or autoimmune uveitis, signifies severe intraocular inflammation or infection. In contrast, lipemic aqueous humor results from the transduction of lipid particles into the anterior chamber due to hyperlipidemia‐induced BAB dysfunction, without significant intraocular inflammation. Its appearance is more homogeneous and milky, and it shows neither gravitational dependence nor association with typical inflammatory signs.

A review of documented reports (Table 1) shows that lipemic aqueous humor predominantly occurs in patients with poorly controlled diabetes mellitus. Diabetes‐related dyslipidemia likely promotes lipid leakage. It alters lipoprotein composition and elevates triglyceride level, prolonging endothelial residence time and enhancing susceptibility to endothelial damage, ultimately contributing to BAB impairment [9, 12]. The fibrin exudate membrane observed on the lens during anterior chamber irrigation in our patient provided direct evidence of BAB dysfunction. Precipitating factors may include poor diet, alcohol consumption, hypothyroidism, nephrotic syndrome, and the use of certain antipsychotic medications such as risperidone [1, 8].

**Table 1:** Available references on lipemic aqueous humor.

Name	Age	Gender	Affected eye	Glucose (mmol/L)	Triglyceride (mmol/L)	The other eye	Past medical history	HbA1c
Robertson and Misch [6]	20	Female	OU	24.8	Lipemic	PDR	Malias, familial hyperlipidemia	19.8%
Qadar et al. [7]	43	Male	OD	/	/	Normal	DM, hypertension, gout, pyelourinary	/
Gopal et al. [8]	32	Male	OD	/	/	Lipemia retinalis	DM and schizophrenic	/
Zhao et al. [1]	39	Male	OD	12.8	15.84	Normal	DM and CRVO	/
Bi et al. [9]	31	Male	OD	14.6	13.55	Normal	DM	12.6
Sun et al. [10]	31	Male	OS	Diabetic ketoacidosis	45.66	/	DM	/
Thanh et al. [11]	44	Male	OS	24.0	38.0	Mild DM change	DM, hypertension, and obesity	/
Our case	26	Female	OS	33.39	23.71	Normal	DM	18.5

This case highlights the need to consider lipemic aqueous humor in the differential diagnosis of anterior chamber opacification, particularly in patients with diabetes and hyperlipidemia. Although no vitreous abnormalities were detected emergently, the initial finding of cocci in blood smears (later deemed contaminants) prompted anterior chamber irrigation and intravitreal antibiotic injection to avoid delaying treatment for possible endophthalmitis. In retrospect, this intervention may have been unnecessary. Emerging evidence indicates that conservative management, including anti‐inflammatory treatment (e.g., corticosteroids) to address the inflammatory component, mydriasis, and strict control of glucose and lipid levels can yield excellent visual and systemic outcomes.

## Author Contributions

J.W.: writing—original draft. J.Y.: writing—review & editing and conceptualization. Y.Z.: writing—review & editing. J.L.: writing—review & editing and conceptualization.

## Funding

No funding was received for this manuscript.

## Disclosure

All authors have read and approved the final version of the manuscript. Jun Li had full access to all data in this study and takes responsibility for the integrity of the data and the accuracy of the data analysis. The supporting source had no involvement in study design; collection, analysis, or interpretation of data; writing of the report; or the decision to submit the report for publication. Jun Li affirms that this manuscript is an honest, accurate, and transparent account of the study being reported; that no important aspects have been omitted; and that any discrepancies have been explained.

## Ethics Statement

The authors have nothing to report.

## Consent

Written informed consent was obtained from the patient for publication of this case report and any accompanying images.

## Conflicts of Interest

The authors declare no conflicts of interest.

## Data Availability

All data generated or analyzed during this study are included in this published article.
